# Playground equipment-related extremity fractures in children presenting to US emergency departments, 2006–2016

**DOI:** 10.1186/s40621-020-00275-w

**Published:** 2020-09-21

**Authors:** Ashley Blanchard, Ava Hamilton, Guohua Li, Peter S. Dayan

**Affiliations:** 1 Department of Emergency Medicine, Columbia University Vagelos College of Physicians and Surgeons, 3959 Broadway, CHN-1-116, New York, NY 10032 USA; 2 Department of Epidemiology, Mailman School of Public Health, Columbia University, 722 West 168th Street #724, New York, NY 10032 USA; 3 Department of Anesthesiology, Vagelos College of Physicians and Surgeons, Columbia University, 722 West 168th Street, Rm 524, New York, NY 10032 USA

**Keywords:** Playground, Fracture, Extremity, Monkey bar, Climbing gym, Equipment

## Abstract

**Background:**

Despite updated playground equipment and improved industry standards, playgrounds remain a common source of childhood injury. Fractures account for 35% of all playground injuries presenting to emergency departments (EDs). We aimed to examine the time trends and epidemiologic patterns of playground equipment-related extremity fractures in children in the United States.

**Methods:**

We analyzed data from the National Electronic Injury Surveillance System. Children ≤14 years presenting to US emergency departments from 2006 to 2016 with playground equipment-related injuries were included. We used weighted complex survey analysis to describe the epidemiologic patterns and severity of playground equipment-related extremity fractures and Joinpoint linear weighted regression analysis to determine trends in extremity fractures.

**Results:**

An annual average of 72,889 children were treated in US EDs for playground equipment-related extremity fractures, yielding a national annual incidence rate of 119.2 per 100,000 children. Playground equipment-related extremity fractures accounted for 33.9% of ED presentations and 78.7% of hospitalizations for playground equipment-related injuries. Of patients with playground equipment-related extremity fractures, 11.2% had severe fractures requiring hospitalization. The annual rate of ED visits due to playground equipment-related extremity fractures remained stable (annual rate of change = 0.74, *p* = 0.14) from 2006 to 2016. Adjusted for age, injuries on monkey bars or climbing gyms were associated with significantly increased odds of extremity fractures in comparison to injuries from other playground equipment (adjusted odds ratio [aOR]: 2.0; 95% CI: 1.9–2.1). Overall, 49.8% of extremity fractures and 54.7% of severe extremity fractures (i.e. those requiring hospitalization) occurred on monkey bars or climbing gyms.

**Conclusions:**

Despite enhanced playground safety standards, national rates of playground equipment-related extremity fractures have remained stable in the US. Extremity fractures remain the most common type of playground injury presenting to EDs and most commonly occur on monkey bars and climbing gyms.

## Background

Playgrounds provide a protected space for children to play and have known physical and cognitive benefits (Trembley et al. 2015). Each year, playground injuries are responsible for approximately 200,000 United States (US) emergency department (ED) visits in children younger than 18 years (Vollman et al. 2009). Since 2006, the overall annual rate of playground injuries have increased significantly (Adelson et al. 2018). Extremity injuries are the most frequent type of playground equipment-related injury, and the majority of injuries to the extremities for which children present to the ED are fractures (Vollman et al. 2009; Loder 2008; Phelan et al. 2001; Norton et al. 2004; Waltzman et al. 1999). Of all playground fractures, 70–90% are fractures of the upper extremity (Ball et al. 1991; Tinsworth et al. 2001). While most fractures can be evaluated and treated in the ED, extremity fractures are the most common reason children presenting to EDs for playground-related injuries require hospital admission (Tinsworth et al. 2001; Adelson et al. 2018). Some fracture types, such as supracondylar fractures of the humerus, may require anesthesia, reduction, and hospitalization (Farnsworth et al. 1998).

ASTM International (ASTM) voluntary standards and US Consumer Product Safety Commission guidelines for playground equipment and surfacing, in homes and public spaces, have primarily focused on how modifiable playground factors impact head injuries. The minimum requirements for the impact attenuation of playground surfacing materials established by ASTM F1292 are determined by simulating the impact of a child’s head with the surface (ASTM F1292–18). It remains unclear if conformance with these surfacing specifications and updates in playground equipment and surfaces have resulted in fewer or less severe extremity fractures (ASTM F1487–17; Mathison and Agrawal. 2010; Sherker and Ozanne-Smith).

In addition to playground surfacing, equipment type and fall height are known to influence the type and severity of extremity injuries (Chalmers et al. 1996; Mott et al. 1997; Laforest et al. 2001). Of the various equipment types, monkey bars or climbing gyms are thought to account for the highest proportion of playground-related fractures (Waltzman et al. 1999; Farnsworth et al. 1998). An estimated 60% of injuries from monkey bars or climbing gyms are fractures and, of those fractures, supracondylar fractures make up the largest proportion (Waltzman et al. 1999). This relationship between monkey bars or climbing gyms and extremity fractures has not been explored since the initial introduction of ASTM standards in the 1990s (Waltzman et al. 1999). As playground equipment has changed over the past 10 years, it is unclear if monkey bars or climbing gyms continue to be a common source of extremity fractures.

Although recent studies have investigated overall playground-related injuries and specifically playground-related traumatic brain injury, no studies have examined recent US playground equipment-related extremity fracture trends and the specific factors associated with these injuries (Adelson et al. 2018; Cheng et al. 2016). It is important to understand if current equipment and surfacing standards have resulted in fewer or less severe extremity fractures (ASTM, 2017, ASTM, 2018, US Consumer Product Safety Commission, 2016). We aimed to examine the time trends and epidemiologic patterns of playground equipment-related extremity fractures in children seen in US emergency departments from 2006 to 2016 (ASTM, 2018). We also assessed the association between different playground equipment types, specifically monkey bars and climbing gyms, and extremity fracture rates overall and extremity fractures that require hospitalization (ASTM, 2017). We hypothesized that national rates of playground equipment-related extremity fracture were increasing, in parallel to increases in overall playground injuries. We also hypothesized that, despite policies to update playground equipment, monkey bars and climbing gyms continue to be associated with significantly increased odds of extremity fractures overall and those requiring hospitalization in children.

## Methods

### Data source

We conducted a cross-sectional study of playground equipment-related extremity fractures presenting to US EDs in children aged ≤14 years using the National Electronic Injury Surveillance System (NEISS) from January 1, 2006 through December 31, 2016. The NEISS is operated by the Consumer Product Safety Commission and collects data on injuries that present to US hospital-based EDs (US Consumer Product Safety Commission). The NEISS samples approximately 100 hospitals to represent a stratified probability sample of the over 5000 US EDs. The EDs included have a minimum of 6 beds, are 24-h EDs, and range in hospital size and type. Coordinators in each NEISS site collect and record specific variables and create a narrative for each patient seen in the ED for an injury during the initial visit. The NEISS variables include primary diagnosis, body part affected, type of injury, disposition, and location of event. The NEISS reports the primary visit diagnosis and primary body part injured.

Cases of playground equipment-related extremity injury were identified using the following inclusion criteria. First, all cases of playground-related injuries in children aged ≤14 years associated with playground equipment were identified using the six NEISS playground equipment product codes. Subsequently, we selected patients with extremity injuries using the NEISS body part codes. To determine the completeness of our search strategy and identify possible missed cases of playground equipment-related extremity fractures, we queried the narratives of all patients with extremity fractures in the NEISS database who did not have associated playground product codes. We queried the narratives for key words related to playground location and equipment (including playground, slide, monkey bar, seesaw, jungle gym, teeter-totter). By identifying cases via equipment codes, we excluded injuries unrelated to playground equipment, such as falls from standing or sports related injuries. Shoulder injuries were excluded, as the mechanism of these fractures is often different from that of other arm injuries.

### Variables

We examined body part injured, setting of injury, equipment type, and disposition. Age was categorized based on developmental age groups and body part injured was grouped into lower and upper extremity injuries. Upper extremity injuries included the NEISS codes for upper and lower arm, elbow, wrist, finger, or hand (the NEISS body part codes 80, 33, 32, 34, 82, 92, respectively). Lower extremity injuries included the NEISS codes for foot, knee, ankle, upper and lower leg, and toe (the NEISS body part codes 83, 35, 37, 81, 36, 93, respectively). We categorized setting of injury as place of recreation, home, school or daycare, or other public property. An injury was determined to occur on playground equipment if it was associated with one of the NEISS product codes related to playground equipment. These equipment categories include slides or sliding boards, seesaws or teeterboards, monkey bars or climbing gyms (including playground gyms or other climbing apparatus), swings or swing sets, other playground equipment, and unspecified playground equipment (the NEISS codes 1242, 1243, 1244, 3246, 3219, 3273 respectively). To differentiate monkey bar injuries from other climbing gym injuries, we searched the narratives of all cases of extremity fractures associated with the NEISS product code for monkey bars or climbing gym for the terms “monkeybars” or “monkey bars”.

We combined NEISS disposition codes to categorize disposition from the ED as discharged (treated and released), admitted (treated and admitted, treated and transferred to another hospital, or held for observation) or other (left against medical advice, left without being seen, died, or unknown). Severe extremity fractures were defined as fractures requiring hospital admission.

### Analyses

We used the NEISS sample weighting based on the inverse probability of selection. Sample weights were analyzed via complex survey procedure to obtain weighted average annual numbers of national ED visits and 95% confidence intervals (CIs). US Census Bureau population estimates for children ≤14 years were used to determine national rates of injury. We performed descriptive analyses using SPSS 24.0 (IBM corporation Armonk, NY) and trend analysis using Joinpoint (Statistical Methodology and Applications Branch, Surveillance Research Program, National Cancer Institute, Bethesda, MD) linear weighted regression. For all analyses, an overall unweighted count of < 20 cases or a weighted estimate < 1200 were considered possibly unstable and noted when reported. A logistic regression was performed, accounting for the complex weighted survey design, to determine the association of equipment type with extremity fracture, adjusting for age as a known confounder.

## Results

### Injury characteristics

**Fig. 1:**
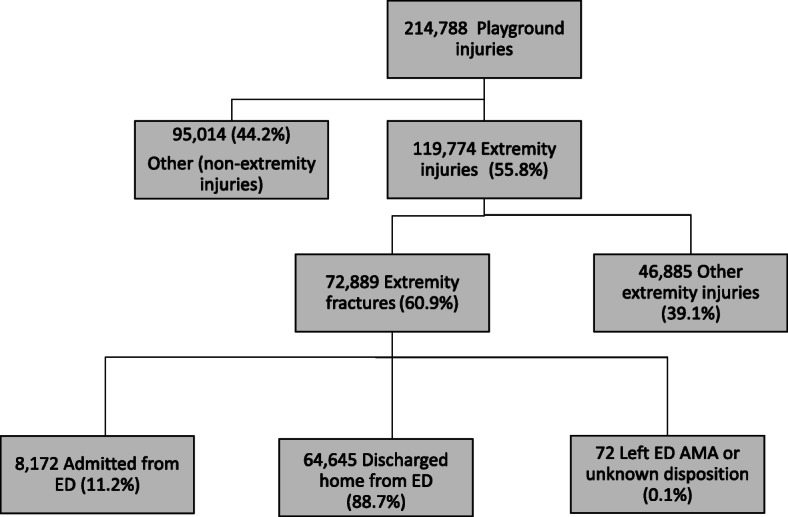
Average annual ED visits for playground equipment-related injuries, extremity fractures, and ED disposition from 2006 to 2016

An annual average of 214,788 children were treated in US EDs for playground equipment-related injuries between 2006 and 2016 (Table 1). Of these injuries, 72,889 (33.9%) were playground equipment-related extremity fractures, with the majority being fractures of the upper extremity (87.4%) (Fig. 1). Nearly half of all playground equipment-related extremity fractures occurred in boys (54.3%) and children aged 5–9 years had the highest rates of playground equipment-related extremity fractures and other playground equipment-related injuries. Most commonly, extremity fractures occurred at places of recreation (35.1%), followed by schools or daycare (29.0%).

**Table 1:** Average annual ED visits for playground-related extremity fractures and other playground-related injuries

	Total			Playground related extremity fracture			Other (non-extremity fracture) playground injuries		
	n	%	95% CI	n	%	Rate per 100,000 (95% CI)	n	%	Rate per 100,000 (95% CI)
Total	214,788	100.0		72,889	100.0	119.2 (95.7–142.8)	141,899	100.0	232.1 (188.7–275.5)
**Age, y**									
0–4 years	60,053	28.0	59,282.0–60,824.0	14,979	20.6	74.1 (56.3–91.9)	45,074	31.8	222.9 (173.1–272.6)
5–9 years	120,904	56.3	120,303.4–121,504.6	48,728	66.9	240.7 (194.7–286.7)	72,176	50.9	356.6 (292.2–420.9)
10–14 years	33,831	15.8	32,997.2–33,831.0	9182	12.6	44.4 (36.3–52.6)	24,649	17.4	119.3 (100.2–138.4)
**Gender**									
Male	114,661	53.4	114,040.8–115,281.2	39,575	54.3	150.1 (120.2–179.9)	75,086	52.9	284.7 (230.5–338.9)
**Equipment Type**									
Monkey bars or climbing gyms	79,157	36.9	78,435.2–79,878.8	36,296	49.8	59.4 (46.7–72.1)	42,861	30.2	70.1 (55.8–84.5)
Swings	48,655	22.7	47,856.1–49,453.9	12,960	17.8	21.2 (17.5–24.9)	35,695	25.2	58.4 (49.1–67.7)
Slides	45,827	21.3	45,021.3–46,632.7	13,904	19.1	22.7 (18.6–26.9)	31,923	22.5	52.2 (42.4–62.1)
Seesaws	3813	1.8	2912.7–4713.3	^b^677	0.9	1.1 (0.9–1.4)	3136	2.2	5.1 (4.1–6.1)
Other playground equipment	16,918	7.9	16,046.1–17,789.9	3884	5.3	6.4 (4.2–8.5)	13,034	9.2	21.3 (14.8–27.9)
Playground equipment not specified	20,418	9.5	19,553.9–21,282.1	5168	7.1	8.5 (5.3–11.6)	15,250	10.7	24.9 (16.5–33.4)
**Setting of injury**									
Place of recreation	73,048	34.0	72,310.1–73,785.9	25,565	35.1	41.8 (32.5–51.1)	47,483	33.5	77.7 (62.2–93.1)
School or daycare	61,048	28.4	60,279.5–61,816.5	21,154	29.0	34.6 (25.2–44.0)	39,894	28.1	65.3 (49.5–81.1)
Home	29,118	13.6	28,273.4–29,962.6	8725	12.0	14.3 (10.8–17.7)	20,393	14.4	33.4 (26.1–40.6)
Other public property	7183	3.3	6290.0–8076.0	2240	3.1	3.7 (2.2–5.1)	4943	3.5	8.1 (5.9–10.3)
Not recorded	44,391	20.7	43,581.9–45,200.1	15,205	20.9	24.9 (17.1–32.6)	29,186	20.6	47.7 (32.4–63.1)
**Disposition**^a^									
Discharged	202,792	94.4	202,577.3–203,006.7	64,645	88.7	105.8 (84.9–126.7)	138,146	97.4	226.0 (183.7–268.3)
Admitted	10,377	4.8	9490.8–11,263.2	8172	11.2	13.4 (10.3–16.5)	2206	1.6	3.6 (2.6–4.6)

^a^Excludes the < 1% of patients who left ED against medical advice, died, or had disposition data missing. No fatalities occurred from playground-related extremity fractures, while 15 fatalities occurred from other (non-extremity fracture) playground injuries

^b^Estimates < 1200 are considered possibly unstable

### Trends

**Fig. 2:**
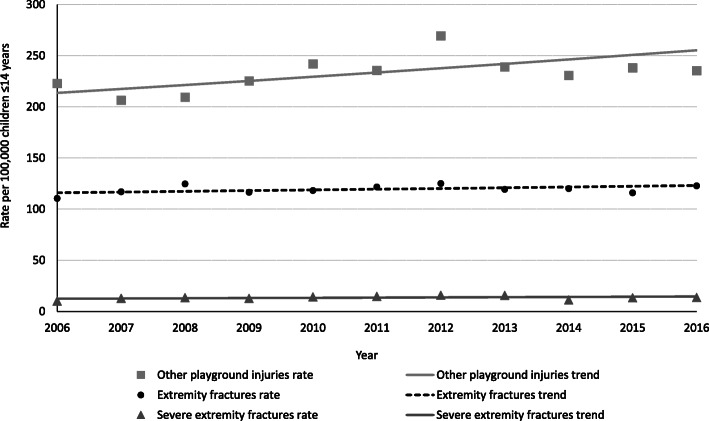
Playground-related extremity fracture rates and all other playground-related injury rates from 2006 to 2016. The playground-related fracture annual modeled rate change (slope) = 0.74 (*p* = 0.14). Other playground-related injuries annual modeled rate change (slope) = 4.05 (*p* = 0.03). Severe playground-related extremity fractures annual modeled rate change (slope) = 0.22 (*p* = 0.08)

The best fitting Joinpoint trend model demonstrated that, from 2006 to 2016, the annual modeled rate change for playground equipment-related extremity fracture remained stable (slope = 0.74; *p* = 0.14) (Fig. 2). The subset of severe playground equipment-related extremity fractures also remained stable from 2006 to 2016 (slope = 0.22; *p* = 0.08). During the same study period, the trend in other playground equipment-related injuries increased (slope = 4.05; *p* = 0.03).

**Fig. 3:**
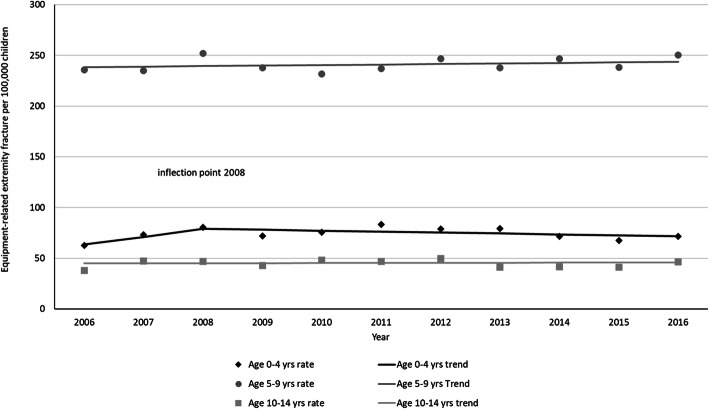
Playground-related extremity fracture rates by age group from 2006 to 2016. For children aged 0–4 years, from 2006 to 2008, annual modeled rate change (slope 1) = 10.7 (p = 0.07) for children aged 0–4 years and from 2008 to 2016, annual modeled rate change (slope 2) = − 0.8 (*p* = 0.25). For children aged 5–9 years, annual modeled rate change (slope) = 0.55 (*p* = 0.47). For children aged 10–14 years, annual modeled rate change (slope) = 0.13 (*p* = 0.77)

When analyzed by age group (Fig. 3), the estimated rate trend in playground equipment-related extremity fractures for children 0–4 years increased from 2006 to 2008 but did not reach statistical significance (slope = 10.7; *p* = 0.07); the rate remained stable subsequent to 2008 (slope = − 0.8; *p* = 0.25). The estimated rate change in playground equipment-related extremity fractures remained stable throughout the study period in children aged 5–9 years (slope = 0.55; *p* = 0.47) and in children 10–14 years (slope 0.13; *p* = 0.77).

The equipment type associated with the injury and the body part injured varied by age (Table 2). Children aged 0–4 years were most commonly injured on slides (37.6%) and most frequently fractured the lower arm (28.7%) or elbow (18.2%). Children aged 5–9 were most commonly injured on monkey bars or climbing gyms (58.5%) and most frequently fractured the lower arm (41.2%) or wrist (23.5%). Children aged 10–14 years sustained the fewest playground equipment-related extremity fractures and were also most commonly injured on monkey bars or climbing gyms (32.7%) and swings (32.6%).

**Table 2:** National average annual estimates of playground equipment-related extremity fractures by age group

Characteristics	0–4 years		5–9 years		10–14 years	
	n (%)	95% CI	n (%)	95% CI	n (%)	95% CI
Total	14,979 (100.0)	11,446–18,513	48,728 (100.0)	39,417–58,040	9182 (100.0)	7562–10,801
**Equipment type**						
Monkey bars or climbing gyms	4782 (31.9)	3468–6097	28,510 (58.5)	22,550–34,470	3005 (32.7)	2377–3632
Slides	5639 (37.6)	4447–6831	6718 (13.8)	5580–7857	1547 (16.8)	1196–1898
Swings	2143 (14.3)	1660–2626	7824 (16.1)	6420–9227	2993 (32.6)	2520–3466
Seesaws	186 (1.2)	124–248	362 (0.7)	270–454	129 (1.4)	69–188
Other playground equipment	875 (5.8)	408–1343	2276 (4.7)	1649–2903	732 (8.0)	432–1033
Playground equipment, not specified	1354 (9.0)	817–1891	3039 (6.2)	1883–4194	775 (8.4)	499–1052
**Body part injured**^a^						
Lower arm (ulna, radius)	4297 (28.7)	3261–5333	20,056 (41.2)	15,714–24,398	3117 (33.9)	2342–3891
Wrist	1932 (12.9)	1497–2367	11,453 (23.5)	9172–13,734	2304 (25.1)	1863–2745
Elbow	2721 (18.2)	1931–3512	8026 (16.5)	6172–9880	614 (6.7)	474–755
Upper arm (humerus)	1790 (11.9)	1042–2537	4692 (9.6)	3414–5970	563 (6.1)	421–706
**Location (setting of injury)**						
Place of recreation	6423 (42.9)	4832–8015	16,132 (33.1)	12,600–19,664	3009 (32.8)	2366–3653
School	2064 (13.8)	1212–2917	16,370 (33.6)	12,023–20,718	2720 (29.6	2036–3403
Other public property	657 (4.4)	432–882	1366 (2.8)	748–1983	218 (2.4)	111–324
Home	2392 (16.0)	1708–3077	5010 (10.3)	3788–6233	1322 (14.4)	947–1698
Location not recorded	3443 (23.0)	2276–4609	9850 (20.2)	6800–12,900	1912 (20.8)	1277–2548
**Disposition**^b^						
Discharged	13,302 (88.8)	10,233–16,370	42,932 (88.1)	34,618–51,245	8412 (91.6)	6927–9897
Admitted	1656 (11.1)	1118–2194	5750 (11.8)	4504–6997	765 (8.3)	573–958

^a^ Only including four most commonly injured body parts

^b^ Excludes those who left the ED against medical advice and those for whom disposition not recorded

### Disposition

While most patients with playground equipment-related extremity fractures were discharged from the ED, 11.2% of children with playground equipment-related extremity fractures were admitted to the hospital for their fractures (Table 1). Children admitted to the hospital with extremity fractures accounted for 78.7% of all admissions for playground equipment-related injuries. The elbow was the most common fracture location in patients admitted to the hospital (31.7%), while only accounting for 15.6% of playground equipment-related fractures.

### Equipment type

Overall, monkey bars or climbing gyms accounted for 49.8% of all extremity fractures and 54.7% of all severe extremity fractures. When controlling for age, monkey bars or climbing gym injuries were 2 times as likely to result in a playground equipment-related extremity fracture (aOR 2.0; 95% CI 1.9–2.1) in comparison to other equipment types. In our narrative review that separated monkey bars and climbing gyms based on the injury description, 81.5% of monkey bar or climbing gym extremity fractures occurred specifically on the monkey bars. In a model using the narrative categories of monkey bars and climbing gyms, controlling for age, monkey bars were 2.4 times more likely to result in a playground equipment-related extremity fracture (aOR 2.4; 95% CI 2.2–2.5), while climbing gyms had a similar risk of extremity fracture (aOR 1.1 95% CI 1.0–1.2) compared to other playground equipment.

Monkey bar or climbing gym injuries were also associated with an increased risk of severe extremity fractures (aOR 1.9; 1.7–2.1) in comparison to other equipment types, when adjusting for age. Using the equipment narratives, a secondary analysis demonstrated that monkey bars were 2.1 times more likely to result in a severe extremity fracture (aOR 2.1; 95% CI 1.9–2.3) and climbing gyms were 1.3 times more likely to result in a severe extremity fracture (aOR 1.3; 95% CI 1.1–6.7) compared to other equipment.

Through narrative review of all cases of extremity injuries that were not associated with playground equipment codes (*n* = 631,981 unweighted cases), we identified 337 unweighted cases of extremity fractures with playground equipment-related terms in the narrative (ASTM, 2017). These case narratives were manually reviewed and 48 unweighted cases (representing 1144 weighted cases) were determined to be missed playground equipment-related extremity fractures (ASTM, 2017). These 48 cases were not included in our analysis. The remaining cases were injuries due to sports, falls from standing or other events unrelated to playground-equipment.

## Discussion

In this large, national database study of playground equipment-related extremity injuries in children ≤14 years, we identified trends and epidemiologic patterns of playground equipment-related extremity fractures presenting to US EDs. Although playground surfacing and equipment standards continue to evolve, we found that extremity fractures remain the most common playground equipment-related injury presenting to EDs, with no meaningful change in trends from 2006 to 2016. These unchanged rates of playground equipment-related extremity fractures occur in the setting of increased rates of overall playground injuries that are well described in previous literature and may be partially attributable to increased concussion awareness (Adelson et al. 2018; Cheng et al. 2016).

While no recent studies have investigated trends in playground equipment-related extremity fractures, our estimate that approximately one third of ED visits for playground injuries were extremity fractures is consistent with prior research (Vollman et al. 2009; O’Brien 2009). Extremity fractures continue to account for the largest proportion of hospital admissions for playground equipment injuries, with no decrease in rates of admission for such fractures. Although the NEISS data do not provide specific details, hospital admissions for playground equipment-related extremity fractures may require operative interventions or monitoring (Waltzman et al. 1999). Prior literature suggests that 40% of extremity fractures that occur on monkey bars are supracondylar fractures, or fractures of the upper arm just above the elbow, and often require surgical intervention (Waltzman et al. 1999; Farnsworth et al. 1998).

Several factors may contribute to the consistent, unchanged rates in playground equipment-related extremity fractures. ASTM playground standards use the maximal fall height from which a life-threatening head injury is unlikely to occur to determine playground surface and height specifications (ASTM F1487–17). Additionally, existing impact-attenuating surfaces were not designed to minimize playground extremity fractures (Adelson et al. 2018; ASTM F1487–17; Sherker and Ozanne-Smith, 2004; Sherker et al., 2005). The impact of these standards on the biomechanics of extremity fractures remains unclear.

There are relatively few studies that have evaluated specific playground factors associated with extremity fractures (Richmond et al. 2018). In one case-control study regarding playground related-extremity fractures, investigators found that equipment height exceeding 1.5 m (4.92 ft) and fall height exceeding 1 m (3.28 ft) were risk factors for extremity fractures (Sherker and Ozanne-Smith, 2004). Surfaces with higher shock attenuation and surface types made of rubber or synthetic fill are associated with decreased injury risk when compared to lower shock attenuation or non-impact absorbing surfaces (Chalmers et al. 1996; Laforest et al. 2001; Sherker et al., 2005). Removing and replacing substandard playground equipment has also been shown to be an effective strategy to prevent playground injuries (Howard et al. 2005). The consistent trend in equipment-related extremity fractures presented in our study may reflect low compliance with existing standards, a lack of playground equipment maintenance, limited awareness of existing interventions, or a need to further determine efficacious interventions.

Both extremity fractures overall and severe extremity fractures were associated with monkey bars. Recent data suggest that monkey bars and climbing gyms are associated with approximately 35% of all playground equipment injuries, with the present study adding that monkey bars are associated with a significant increased risk of extremity fractures overall and severe extremity fractures (O’Brien 2009; Consumer Product Safety Commission Public Playground Safety Handbook 2015). The risk associated with monkey bars may represent a greater exposure to this equipment type, an increase in equipment risk, or specific fall biomechanics from monkey bars. Prior data report falls from any equipment type are more likely to result in fractures compared to other injury mechanisms, and falls account for 90% of monkey bar injuries (Consumer Product Safety Commission Public Playground Safety Handbook 2015; Fiissell et al. 2005). Monkey bars likely represent an equipment that poses a high risk for falls from a height and a mechanism that predisposes children to extremity fractures.

While it is important to provide developmentally challenging and appealing playground equipment, extremity fractures and extremity fractures requiring admission and potentially surgical intervention should be minimized (Fiissell et al. 2005). Current playground standards and prevention efforts appear insufficient to significantly decrease rates of playground equipment-related extremity fractures. This study illustrates the need to develop and disseminate novel or updated playground standards that directly address extremity fractures.

This study had several limitations which should be noted. Our national estimates likely underestimate the rates of playground equipment-related extremity fractures. The NEISS collects data only for the most severe injury at ED presentation, so our analysis does not include playground equipment-related extremity fractures associated with other more severe injuries. The data are also limited to patients seen in EDs. Playground-equipment related extremity fractures evaluated in primary care or urgent care clinics were not included in our study. In addition, playground equipment types and locations of injuries are confined to existing NEISS codes. For example, daycare and school injuries are analyzed together based on these codes. The product code for monkey bars and climbing gyms consist of a heterogenous group of playground equipment types. Our narrative search and subset analysis was used to address this limitation, but may not fully account for misclassification. It is also important to note that we did not assess seasonality of injuries. Playground exposure changes in relation to the school year and the seasons. Our study did not explore these injury patterns. Finally, due to the retrospective nature of the NEISS data collection, our analysis does not include the amount of time children are exposed to specific equipment types. This limited our ability to determine if equipment specific rates of injuries were related to greater exposure or to inherent risk of specific playground equipment.

## Conclusion

Despite updated playground standards and surfacing, this nationally representative study of playground equipment-related extremity fractures demonstrates the incidence of playground equipment-related extremity fractures has remained stable over the past 11 years. Monkey bars are most commonly associated with playground equipment-related extremity fractures and, more concerningly, severe extremity fractures. Strategies and improvements in playground standards, design, surfacing, and maintenance are needed to minimize the risk of extremity fractures and severe extremity fractures. Future studies should further investigate height and surfacing standards aimed to specifically prevent playground equipment-related extremity fractures.

## Data Availability

The datasets generated and/or analyzed during the current study are available in the NEISS repository, https://www.cpsc.gov/Research%2D%2DStatistics/NEISS-Injury-Data.

## Abbreviations

US: United States
ED: Emergency department
ASTM: ASTM International
NEISS: National Electronic Injury Surveillance System
aOR: Adjusted odds ratio
